# Comparative Outcomes of Hip Fractures in Centenarians Compared to Non-Centenarians

**DOI:** 10.3390/jcm15208180

**Published:** 2026-10-21

**Authors:** Hüseyin Bilgehan Çevik, Ahmet Acar, Ahmet Berkay Girgin, Gözde Şengül Ayçiçek, Meltem Koca, Osman Yağız Atlı, Ömer Torun, Evrim Duman

**Affiliations:** 1 Department of Orthopedics and Traumatology, Ankara Etlik City Hospital, University of Health Sciences, 06170 Ankara, Türkiye; 2 Department of Orthopedics and Traumatology, Akyurt State Hospital, 06750 Ankara, Türkiye; 3 Department of Geriatrics, Ankara Etlik City Hospital, University of Health Sciences, 06170 Ankara, Türkiye

**Keywords:** hip fracture, centenarians, mortality, ASA score, survival

## Abstract

**Background/Objectives**: The number of hip fractures among centenarians is rising, yet their outcomes and the factors influencing mortality remain inadequately characterized. This study compared postoperative outcomes between centenarians and non-centenarians and examined perioperative factors associated with mortality. **Methods**: This retrospective cohort study included 31 centenarians (≥100 years) who underwent hip fracture surgery between October 2022 and March 2024 and 176 consecutive non-centenarians (65–99 years) admitted between October 2022 and April 2023. Associations between centenarian status, American Society of Anesthesiologists (ASA) score, comorbidity-only Charlson Comorbidity Index (CCI), surgical procedure, and time to surgery with 30-day and 12-month mortality were analyzed using logistic regression with clinically pre-selected covariates (Firth’s method for 30-day mortality). **Results**: Centenarians exhibited higher ASA scores, while CCI values did not differ between groups. Centenarians experienced longer intensive care unit and hospital stays. The complication rate was numerically higher among centenarians (35.5% vs. 30.1%), although this difference was not statistically significant (*p* = 0.55). Thirty-day mortality was 29.0% for centenarians compared to 6.2% for non-centenarians, and 12-month mortality was 80.6% versus 30.1% (both *p* < 0.001). Centenarian status (30-day odds ratio [OR] 4.87, 95% confidence interval [CI] 1.73–13.60; 12-month OR 8.19, 95% CI 3.16–24.22) and higher ASA scores were associated with increased mortality, whereas CCI was not. Longer time to surgery was associated with higher 12-month mortality (OR 1.23 per 24 h, 95% CI 1.01–1.51). **Conclusions**: Centenarians demonstrated substantially higher short- and long-term mortality following hip fracture surgery compared to younger older adults. Extreme age and elevated ASA scores were associated with increased mortality, although unmeasured factors such as frailty may contribute to these associations. Hip fracture in centenarians should be considered a major life-limiting event that necessitates early goals-of-care discussions.

## 1. Introduction

The population aged over eighty is projected to triple by 2050 [1], and the global number of centenarians is expected to exceed 25 million by 2100 [2]. As the number of older individuals increases, the incidence of hip fractures at advanced ages is also expected to rise [3]. Consequently, the number of centenarians experiencing hip fractures will likely continue to increase, presenting a significant clinical and economic challenge for healthcare systems.

Centenarians, defined in the demographic and gerontological literature as individuals aged 100 years or older [2,4], are not simply an extension of the octogenarian or nonagenarian population. They represent a highly selected group of survivors, many of whom have delayed or escaped the major age-related diseases [4]. Consistent with this, centenarians with hip fracture have been reported to have a lower comorbidity burden than younger patients with hip fracture [5,6], yet higher postoperative mortality [6,7]. Outcome data and risk estimates derived from younger older adults may therefore not be directly applicable to centenarians, and this population warrants separate evaluation in hip fracture research.

Two-year mortality following hip fracture has been reported as approximately 45% in nonagenarians [8] and 70% in centenarians [9]. Functional recovery is also limited in this age group. In a multicenter cohort, only 16% of centenarians were able to walk outdoors one year after surgery [10], and in another series, none of the patients was independent in activities of daily living one month after discharge [11]. Additionally, centenarians experience higher in-hospital mortality than younger patients and are more frequently discharged to nursing homes [7,12].

Although hip fractures in older adults have been extensively studied, data specifically addressing centenarians remain limited, are derived mostly from small retrospective series, and are of moderate to low quality overall [13]. Moreover, few studies have compared centenarians with a contemporaneous group of younger patients treated in the same department under the same perioperative protocol. The aims of this study were therefore twofold: first, to compare postoperative morbidity and short- and long-term mortality between centenarians and non-centenarians undergoing hip fracture surgery in a contemporary cohort and, second, to identify perioperative factors associated with mortality.

## 2. Materials and Methods

### 2.1. Study Design and Patients

This retrospective cohort study was performed in the Department of Orthopedics and Traumatology at Ankara Etlik City Hospital, a tertiary referral center, utilizing data extracted from the hospital’s electronic records. Institutional ethics approval (AESH-BADEK-2024-929) was secured prior to data collection, and the study adhered to the principles outlined in the Declaration of Helsinki.

**Figure 1:**
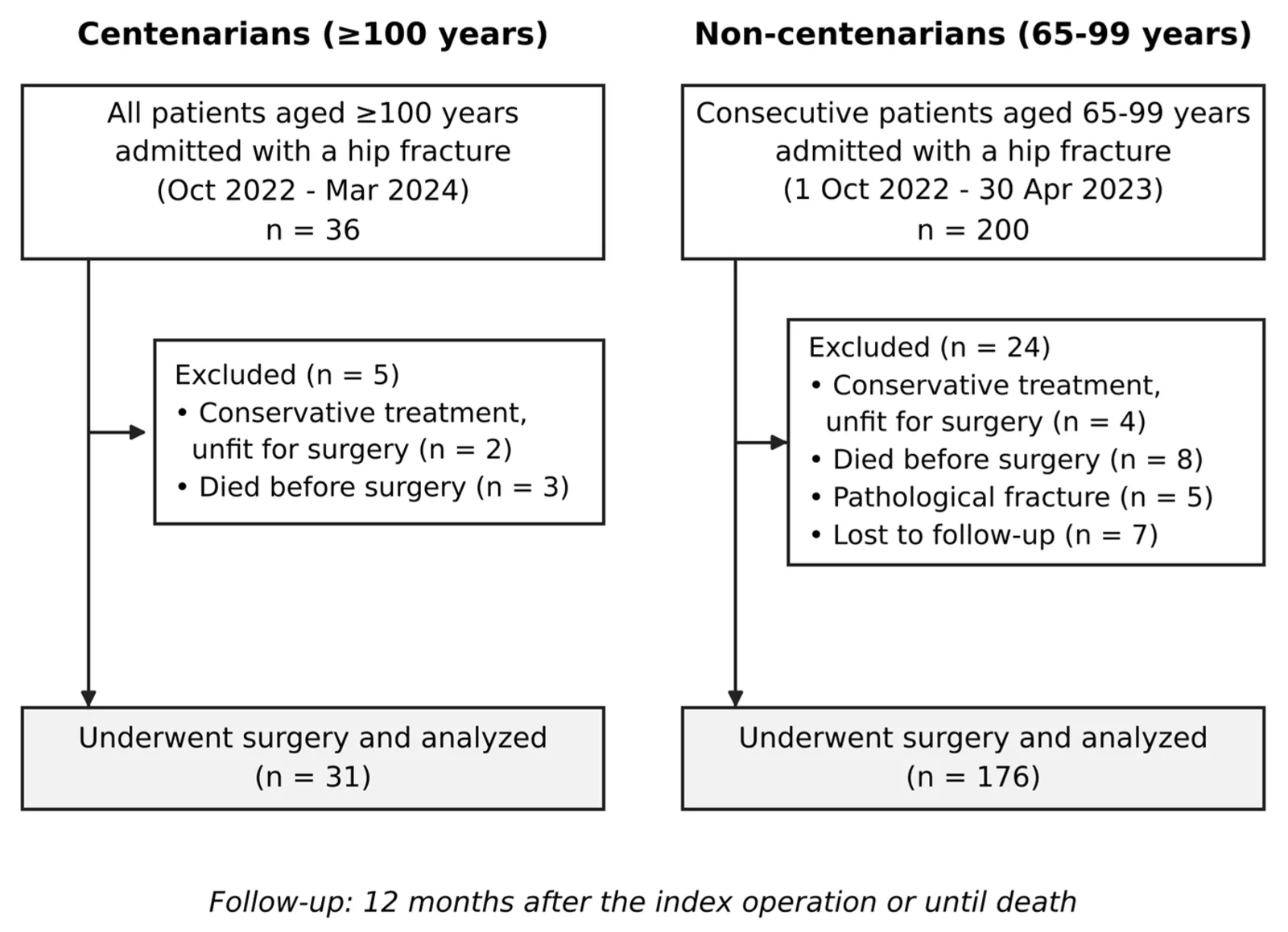
Flowchart of patient selection. All patients screened had been admitted with a hip fracture; patients treated conservatively, those who died before surgery and those meeting the other exclusion criteria were excluded. The comparison group comprised the first 200 consecutive patients aged 65–99 years admitted from 1 October 2022 to 30 April 2023.

Patients admitted with a proximal femoral fracture (femoral neck, including basicervical, pertrochanteric, or subtrochanteric fracture) between October 2022 and March 2024 were screened. All patients aged 100 years or older admitted during this period were screened for the centenarian group (*n* = 36). For the comparison group, the first 200 consecutive patients aged 65 to 99 years admitted with a hip fracture from 1 October 2022 onward were screened (admission dates 1 October 2022 to 30 April 2023); the size of this group was set at 200 without a formal sample size calculation. In total, 982 patients with hip fracture were treated in the department during the study period. Eligibility criteria included undergoing surgical treatment (internal fixation or arthroplasty). Exclusion criteria were conservative management due to being medically unfit for surgery, death before surgery, pathological fracture resulting from a primary or metastatic bone tumor, previous surgery involving the same hip, incomplete preoperative imaging or clinical data, or unavailable 12-month vital status. After applying these criteria, 31 centenarians and 176 non-centenarians were included in the analysis (Figure 1).

### 2.2. Data Collection and Definitions

The collected variables comprised age, sex, comorbidities, fracture type (femoral neck fracture, including basicervical fractures classified as AO/OTA 31B3, or extracapsular fracture), surgical procedure (internal fixation or arthroplasty), American Society of Anesthesiologists (ASA) score, time from admission to surgery (hours), postoperative intensive care unit (ICU) length of stay, hospital length of stay (from admission to discharge), and postoperative complications. Comorbidity burden was assessed using the original Charlson Comorbidity Index (CCI) [14], calculated from documented comorbid conditions without inclusion of age points. The age-adjusted version of the index [15] was not applied, as age, specifically centenarian status, was analyzed as a separate variable. The number of comorbidities was determined from the same documented conditions.

The choice of surgical procedure was determined by the treating surgeon based on fracture type and patient-specific factors. Femoral neck fractures, including basicervical fractures, were typically managed with hemiarthroplasty or total hip arthroplasty, whereas extracapsular (pertrochanteric and subtrochanteric) fractures were treated with internal fixation using either a proximal femoral nail or a dynamic hip screw. Postoperative ICU admission was determined on a case-by-case basis by the attending anesthesiologist, as no formal institutional admission criteria existed. All patients adhered to a standardized postoperative protocol, which included weight-bearing as tolerated and mobilization beginning on the first postoperative day whenever feasible.

Postoperative complications were documented from the time of surgery until 12 months postoperatively or until death, utilizing records from the index admission, readmissions, and outpatient visits. Complications were categorized as either medical (delirium, pulmonary embolism, pneumonia, pulmonary edema or heart failure, urinary tract infection, stroke, and pressure ulcer) or surgical (surgical site infection or wound complication; dislocation; and mechanical failure, including cut-out or implant failure, nonunion, and peri-implant or periprosthetic fracture). Complications were recorded on a per-patient basis, with patients experiencing multiple complications counted only once in the overall complication rate. Pre-existing conditions, new fractures at other anatomical sites, contralateral hip fractures, and unrelated surgical procedures were excluded from the definition of complications. Reoperation was defined as any subsequent surgical procedure performed on the operated hip within 12 months.

### 2.3. Outcomes and Follow-Up

The index operation was the first hip fracture operation during the study period that met the eligibility criteria of the respective group; an earlier operation on the contralateral hip, performed before the study period or, in centenarians, before the age of 100 years, was not considered an index operation. The primary outcomes were 30-day and 12-month mortality, defined as death within 30 and 365 days after the index operation, respectively. In both groups, in-hospital mortality was defined as death before discharge from the index admission. Secondary outcomes included postoperative complications, reoperation, and lengths of stay in the intensive care unit (ICU) and hospital. Vital status and dates of death, including deaths occurring outside the hospital, were determined at the time of data collection (October to December 2025) using the hospital information system, which retrieves death records from the national civil registration system (MERNIS). Survival was calculated from the index operation in all patients, and follow-up was not restarted when a contralateral hip fracture was operated on after the index operation. All patients were followed for 12 months after the index operation or until death.

### 2.4. Statistical Analysis

Continuous variables were summarized as mean ± standard deviation (SD) and/or median with interquartile range (IQR), while categorical variables were presented as counts and percentages. Due to non-normal distribution of most continuous variables (Shapiro–Wilk test), group comparisons utilized the Mann–Whitney U test. Categorical variables were compared using Pearson’s chi-square test, or Fisher’s exact test when any expected cell count was below five. Mortality proportions were reported with 95% confidence intervals (CIs) calculated using the Wilson method. Survival during the first postoperative year was estimated by the Kaplan–Meier method with 95% CIs (log-log transformation) and compared using the log-rank test; patients alive at 365 days were censored.

Associations with 30-day and 12-month mortality were evaluated using logistic regression. Covariates were selected a priori based on clinical relevance rather than univariable screening [16]. Due to the occurrence of only 20 deaths within 30 days, the primary 30-day model was restricted to two covariates, centenarian status and ASA score (10 events per variable) [17], and was fitted using Firth’s penalized likelihood method with profile penalized likelihood CIs [18]. The 12-month model (78 deaths) included centenarian status, sex, ASA score, CCI, surgical procedure (arthroplasty versus internal fixation), and time from admission to surgery (per 24 h), corresponding to approximately 13 events per variable. Postoperative variables (complications and ICU and hospital length of stay) were excluded as predictors, as they occur after surgery and may be on the causal pathway between baseline condition and death. Discrimination of the 12-month model was assessed with the C-statistic, lack of fit with the Hosmer–Lemeshow goodness-of-fit test, and multicollinearity with variance inflation factors. As fewer than 10 in-hospital deaths occurred, in-hospital mortality was compared between groups without multivariable modeling.

Sensitivity analyses included: (i) an extended 30-day Firth model incorporating CCI, surgical procedure, and time to surgery; (ii) a Firth version of the 12-month model; (iii) models substituting centenarian status with age as a continuous variable (per 5 years); (iv) an analysis restricted to patients aged 90 years or older, comparing centenarians with nonagenarians; and (v) a secondary 12-month model including postoperative complications. Mortality was also described across age groups (65–69, 70–79, 80–89, 90–99, and ≥100 years) and tested for trend using the Cochran–Armitage test. A two-sided *p*-value less than 0.05 was considered statistically significant. All analyses were conducted using R version 4.3.3 (R Foundation for Statistical Computing, Vienna, Austria) with the survival (version 3.5-8) and logistf (version 1.26.1) packages.

## 3. Results

Among 36 centenarians and 200 consecutive non-centenarians admitted with a hip fracture, 5 centenarians and 24 non-centenarians were excluded for reasons detailed in Figure 1. The final analysis comprised 31 centenarians and 176 non-centenarians, with approximately two-thirds of each group being women. Seven non-centenarians, and no centenarians, underwent surgery for a contralateral hip fracture after the index operation.

Baseline characteristics are presented in Table 1. Centenarians exhibited higher ASA scores compared to non-centenarians (*p* = 0.023), while comorbidity burden was similar between groups (median CCI 1 [IQR 0–2] in both groups, *p* = 0.41; median number of comorbidities 2 in both groups, *p* = 0.59). Femoral neck fractures, including basicervical fractures, were more prevalent among centenarians (61.3%) than non-centenarians (34.1%, *p* = 0.004). Arthroplasty was performed more frequently in centenarians (61.3% vs. 30.7%, *p* = 0.001), corresponding to the higher proportion of femoral neck fractures. All 19 centenarians and 54 of 60 non-centenarians (90.0%) with femoral neck fractures underwent arthroplasty (*p* = 0.33), while all extracapsular fractures were managed with internal fixation. The time from admission to surgery did not differ significantly between groups (median 44.0 vs. 27.5 h, *p* = 0.41).

All centenarians and 164 of 176 non-centenarians (93.2%) were admitted to the ICU following surgery (*p* = 0.22). Centenarians experienced longer ICU stays (median 4.0 [IQR 3.0–5.5] vs. 1.0 [1.0–2.0] days, *p* < 0.001) and longer overall hospital stays (median 14.0 [10.0–16.0] vs. 7.5 [5.0–10.0] days, *p* < 0.001). Hospital length of stay did not differ significantly between patients treated with arthroplasty and those treated with internal fixation, either overall (median 8.0 vs. 8.0 days, *p* = 0.29), within centenarians (14.0 vs. 12.5 days, *p* = 0.57), or within non-centenarians (7.0 vs. 8.0 days, *p* = 0.58).

**Figure 2:**
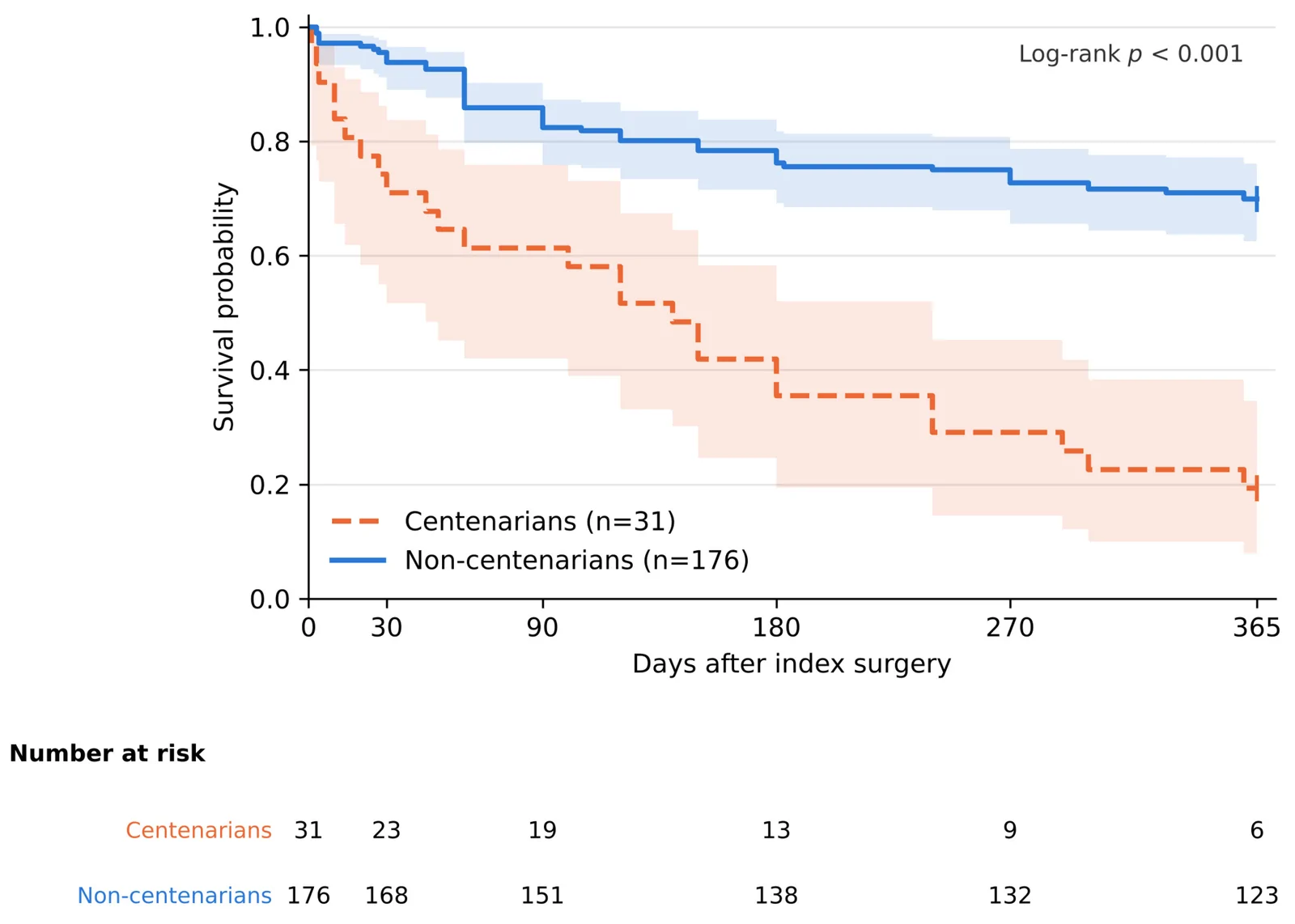
Kaplan–Meier survival curves for centenarians and non-centenarians during the first 12 months after the index hip fracture surgery. Shaded areas indicate 95% confidence intervals; patients alive at 365 days were censored. The numbers of patients at risk are shown below the graph (log-rank *p* < 0.001).

Mortality outcomes are presented in Table 2. In-hospital mortality was higher among centenarians (12.9%, *n* = 4) compared to non-centenarians (2.8%, *n* = 5; *p* = 0.030). Because this comparison was based on few events, it should be interpreted with caution; with one fewer in-hospital death among centenarians, the difference would not have been statistically significant (*p* = 0.10). Thirty-day mortality rates were 29.0% (95% CI 16.1–46.6) for centenarians and 6.2% (95% CI 3.5–10.8) for non-centenarians. Twelve-month mortality was 80.6% (95% CI 63.7–90.8) in centenarians and 30.1% (95% CI 23.8–37.3) in non-centenarians (both *p* < 0.001). Kaplan–Meier analysis indicated lower survival among centenarians throughout the first postoperative year (log-rank *p* < 0.001; Figure 2), with estimated 12-month survival rates of 19.4% (95% CI 7.9–34.6) for centenarians and 69.9% (95% CI 62.5–76.1) for non-centenarians.

In the primary 30-day mortality model (*n* = 207; 20 deaths), centenarian status (odds ratio [OR] 4.87, 95% CI 1.73–13.60, *p* = 0.003) and higher ASA scores (OR 4.03 per point, 95% CI 1.87–9.48, *p* < 0.001) were significantly associated with increased mortality (Table 3). In the 12-month model (*n* = 207; 78 deaths), centenarian status (OR 8.19, 95% CI 3.16–24.22, *p* < 0.001), higher ASA scores (OR 2.64, 95% CI 1.54–4.73, *p* < 0.001), and longer time from admission to surgery (OR 1.23 per 24 h, 95% CI 1.01–1.51, *p* = 0.039) were associated with mortality. Sex, CCI, and surgical procedure were not significantly associated with mortality in this model (Table 3). The 12-month model demonstrated acceptable discrimination (C-statistic 0.75). The Hosmer–Lemeshow test showed no evidence of lack of fit (*p* = 0.94); however, a non-significant result of this test does not by itself establish good calibration. All variance inflation factors were below 1.5.

Sensitivity analyses yielded consistent results (Appendix A, Table A2). In the extended 30-day Firth model, centenarian status (OR 4.83, 95% CI 1.56–15.40) and ASA score (OR 3.48, 95% CI 1.51–8.70) remained significantly associated with mortality, while CCI, surgical procedure, and time to surgery were not. When centenarian status was replaced by age, each 5-year increase in age was associated with higher 30-day (OR 1.37, 95% CI 1.10–1.72) and 12-month (OR 1.48, 95% CI 1.25–1.77) mortality. Among patients aged 65 to 99 years, 30-day mortality ranged from 0% to 7.3% and 12-month mortality from 23.6% to 35.7% across age groups, compared with 29.0% and 80.6% in centenarians (*p* for trend < 0.001 for both; Appendix A, Table A1). For patients aged 90 years or older (*n* = 49), 12-month mortality was 80.6% in centenarians and 27.8% (5 of 18) in nonagenarians (*p* < 0.001; ASA-adjusted OR 6.53, 95% CI 1.70–27.84), while 30-day mortality was 29.0% and 5.6% (*p* = 0.070). In the secondary 12-month model, postoperative complications were associated with mortality (OR 2.61, 95% CI 1.28–5.40, *p* = 0.009), with minimal changes in estimates for other variables.

Within 12 months postoperatively, complications occurred in 11 centenarians (35.5%) and 53 non-centenarians (30.1%), a difference that was not statistically significant (*p* = 0.55; Table 2). Delirium was the most frequently observed complication in both groups. Reoperation within 12 months was required in 1 centenarian (3.2%) and 15 non-centenarians (8.5%) (*p* = 0.48).

## 4. Discussion

In this cohort, hip fracture surgery in centenarians was followed by markedly higher mortality than in non-centenarians, with nearly one-third of centenarians dying within 30 days and four out of five within the first postoperative year. Centenarians also experienced longer ICU and hospital stays, while the overall complication rate was only numerically higher and did not differ significantly between groups. After adjusting for ASA score and comorbidity, centenarian status remained associated with increased mortality. However, as the comparison group was on average 22 years younger, this variable primarily reflects extreme chronological age. Due to the absence of data on frailty, pre-fracture mobility, cognitive status, and nutritional status, the study design cannot distinguish a centenarian-specific effect from the broader impact of advanced age and associated unmeasured factors. A higher ASA score was also independently associated with mortality in the entire cohort; however, the small number of centenarians precluded testing whether this association persisted within the centenarian subgroup.

It remains unclear whether hip fracture increases mortality beyond the already high background mortality at extreme ages. In a Scottish series of patients over 95 years, one-year mortality did not differ significantly from that of the age-matched general population [19]. This study did not include a standardized comparison with the general population, so the excess mortality attributable to the fracture itself cannot be estimated. Within the same institution and under the same perioperative protocol, however, mortality among centenarians was several times higher than among younger patients. Mortality rates were relatively similar across age groups between 65 and 99 years but increased sharply in centenarians. In analyses restricted to patients aged 90 years or older, centenarians still exhibited substantially higher 12-month mortality than nonagenarians. Given that only 18 nonagenarians were included, this comparison is imprecise and should be validated in larger cohorts.

Centenarians had longer ICU and hospital stays, consistent with a previous report of extended acute hospitalizations in this population [20]. However, length of stay is an outcome variable influenced by complications, in-hospital death, and discharge logistics, and its association with mortality varies across settings. Longer hospital stays have been linked to higher post-discharge mortality in New York State [21] and in a multicenter cohort [22], while shorter stays were associated with higher 30-day mortality in Sweden [23]. Consequently, length of stay was not used as a predictor of mortality in these analyses. Additionally, all centenarians and most non-centenarians were admitted to the ICU postoperatively at the discretion of the attending anesthesiologist, without standardized admission criteria. Therefore, the longer ICU stays observed in centenarians reflect both their clinical course and local admission and discharge practices, and should be interpreted cautiously.

Previous studies have documented longer hospital stays following both arthroplasty [24] and internal fixation [22]. In this cohort, hospital length of stay did not differ by surgical procedure, either overall or within each age group, indicating that patient-related factors likely played a greater role than implant type. Arthroplasty was performed more frequently in centenarians due to a higher proportion of femoral neck fractures in this group. After adjustment, surgical procedure was not associated with mortality; however, this comparison was not randomized and may be subject to confounding by indication.

Studies of centenarians with hip fracture have consistently demonstrated high postoperative mortality. Reported one-year mortality rates in operated centenarians have ranged from 33% in a small series [25] to 66% [11]; rates were 39.3% in a multicenter cohort [10] and 54.2% in a single-center series [26], while a meta-analysis of 23 studies estimated a pooled one-year mortality of 53.8% [13]. In a large statewide database, in-hospital mortality was higher in centenarians than in patients aged 81 to 99 and 65 to 80 years (7.4% vs. 4.4% vs. 2.6%) [7]. Similarly, in a German geriatric trauma registry, in-hospital mortality was 15.4% in centenarians compared with 3.6% in patients younger than 80 years [12]. The 12-month mortality observed in this cohort (80.6%) exceeds most previously reported rates. Differences in case mix, surgical selection, small sample size, and the single-center design may contribute to this discrepancy and should be considered when comparing results across studies.

Comorbidity burden and functional status have been frequently evaluated as prognostic factors in centenarians with hip fracture [10]. In one series, a higher CCI was associated with in-hospital mortality, and having more than three comorbidities was linked to 30-day mortality [26]. Other studies identified pre-fracture ambulatory or functional status as the primary predictor of mortality [10,11], even when most patients had low CCI values [11]. In this cohort, the comorbidity-only CCI did not differ between centenarians and non-centenarians and was not associated with mortality. This finding aligns with reports that centenarians with hip fracture have fewer comorbidities than younger patients but experience higher postoperative mortality [5,6]. However, the absence of an independent association for the CCI should not be interpreted as evidence that comorbidity burden is unimportant. The CCI and ASA score capture overlapping aspects of health status [27,28], the CCI was derived retrospectively from documented diagnoses, and the limited sample size reduced the ability to detect modest effects.

In this study, a higher ASA score was independently associated with both 30-day and 12-month mortality. In previous centenarian cohorts, mortality was primarily linked to pre-fracture functional status, dementia, comorbidity, anemia, and male sex, while the ASA grade was not identified as an independent predictor [10,11,26]. These discrepancies may reflect heterogeneity in patient characteristics, case selection, and preoperative assessment. The ASA score represents the anesthesiologist’s overall assessment of physiological status and may capture aspects of frailty and physiological reserve not reflected in comorbidity indices. However, data on frailty, pre-fracture mobility, cognitive status, and nutritional status were not available, so the relative contributions of physiological reserve and comorbidity cannot be determined from this study. In a large registry, the difference in in-hospital mortality between centenarians and patients younger than 80 years decreased after matching for a geriatric risk score [12], suggesting that such factors may explain part of the mortality attributed to extreme age.

A longer interval from admission to surgery was associated with higher 12-month mortality, but not with 30-day mortality. Large population-based studies have linked prolonged waiting times to increased mortality and complication rates [29], and a longer time to surgery predicted one-year mortality in a multicenter cohort [22]. However, time to surgery did not affect in-hospital mortality among centenarians in a statewide database [7]. In a retrospective cohort such as this, the observed association does not imply causality. A longer interval to surgery may indicate medical instability, the need for preoperative optimization (such as reversal of anticoagulation), or other unmeasured clinical factors. Therefore, these results do not establish that delayed surgery itself increased mortality.

This study has several limitations. First, its retrospective design limits causal inference and is subject to selection and information bias. Second, the single-center design may limit the generalizability of our findings to settings with different perioperative care models, including different ICU admission policies. Third, pre-fracture functional status, cognitive impairment, nutritional status and frailty were not systematically available and could not be included in the analyses; these factors may account for part of the associations attributed to extreme age and ASA score. Fourth, the comparison group consisted of the first 200 consecutive patients admitted from 1 October 2022 rather than all non-centenarians treated during the study period, and was therefore recruited during the first 7 months of the 18-month study period; it also included only 18 patients aged 90 to 99 years, which limited the precision of the age-restricted analysis. Fifth, the number of centenarians was small, reflecting the rarity of this population; this resulted in wide confidence intervals, limited the number of covariates in the 30-day model, and precluded multivariable analyses of in-hospital mortality and within the centenarian subgroup.

## 5. Conclusions

Hip fracture surgery in centenarians was associated with significantly higher short- and long-term mortality and with longer ICU and hospital stays compared to younger older adults, while overall complication rates did not differ significantly. Extreme age and a higher ASA score were associated with mortality, although unmeasured factors such as frailty and pre-fracture function may contribute to these associations. These findings underscore the importance of thorough perioperative assessment; appropriate resource allocation; and early, realistic goals-of-care discussions for centenarians with hip fracture, for whom the fracture should be considered a major life-limiting event rather than solely an orthopedic injury.

## Figures and Tables

**Table 1:** Baseline characteristics of centenarians and non-centenarians.

Variable	Centenarians (*n* = 31)	Non-Centenarians (*n* = 176)	*p*-Value
Age, years, mean ± SD	102.1 ± 2.1	80.1 ± 7.5	<0.001
Female sex, *n* (%)	21 (67.7)	112 (63.6)	0.66
ASA score, median (IQR)	3 (3–3)	3 (2–3)	0.023
ASA score ≥ 3, *n* (%)	24 (77.4)	104 (59.1)	0.053
CCI (comorbidity-only), median (IQR)	1 (0–2)	1 (0–2)	0.41
Number of comorbidities, median (IQR)	2 (2–3)	2 (1.8–3)	0.59
Femoral neck fracture (including basicervical), *n* (%)	19 (61.3)	60 (34.1)	0.004
Arthroplasty, *n* (%)	19 (61.3)	54 (30.7)	0.001
Arthroplasty in femoral neck fractures, *n*/N (%)	19/19 (100)	54/60 (90.0)	0.33
Time from admission to surgery, h, median (IQR)	44.0 (17.5–69.0)	27.5 (16.0–58.2)	0.41
Surgery > 48 h after admission, *n* (%)	13 (41.9)	61 (34.7)	0.44
Postoperative ICU admission, *n* (%)	31 (100)	164 (93.2)	0.22
ICU stay, days, median (IQR)	4.0 (3.0–5.5)	1.0 (1.0–2.0)	<0.001
Hospital stay, days, median (IQR)	14.0 (10.0–16.0)	7.5 (5.0–10.0)	<0.001

Values are *n* (%), mean ± standard deviation (SD) or median (interquartile range, IQR). *p*-values were calculated with the Mann–Whitney U test for continuous variables and with Pearson’s chi-square test, or Fisher’s exact test when an expected count was below five, for categorical variables. ASA, American Society of Anesthesiologists; CCI, Charlson Comorbidity Index (original version without age points); ICU, intensive care unit. Hospital stay was measured from admission to discharge or in-hospital death.

**Table 2:** Mortality, postoperative complications and reoperations.

Outcome	Centenarians (*n* = 31)	Non-Centenarians (*n* = 176)	*p*-Value
Mortality, *n* (%; 95% CI)			
In-hospital	4 (12.9; 5.1–28.9)	5 (2.8; 1.2–6.5)	0.030
30-day	9 (29.0; 16.1–46.6)	11 (6.2; 3.5–10.8)	<0.001
12-month	25 (80.6; 63.7–90.8)	53 (30.1; 23.8–37.3)	<0.001
Complications within 12 months, *n* (%)			
Any complication	11 (35.5)	53 (30.1)	0.55
Delirium	4 (12.9)	15 (8.5)	0.50
Pulmonary embolism	1 (3.2)	11 (6.2)	1.00
Pneumonia	0	1 (0.6)	1.00
Pulmonary edema or heart failure	0	2 (1.1)	1.00
Urinary tract infection	2 (6.5)	2 (1.1)	0.11
Stroke	0	1 (0.6)	1.00
Pressure ulcer	0	1 (0.6)	1.00
Surgical site infection or wound complication	1 (3.2)	7 (4.0)	1.00
Dislocation	1 (3.2)	4 (2.3)	0.56
Mechanical failure †	3 (9.7)	11 (6.2)	0.45
Reoperation within 12 months, *n* (%)	1 (3.2)	15 (8.5)	0.48

Mortality was calculated from the index operation; in-hospital mortality refers to death before discharge from the index admission. Complications were counted per patient; a patient could have more than one complication type. *p*-values: Pearson’s chi-square test for 12-month mortality and any complication; Fisher’s exact test for the other rows. 95% CIs were calculated with the Wilson method. † Cut-out or implant failure, nonunion, and peri-implant or periprosthetic fracture.

**Table 3:** Logistic regression analyses of 30-day and 12-month mortality.

Variable	Unadjusted OR (95% CI)	*p*	Adjusted OR (95% CI)	*p*
(A) 30-day mortality (*n* = 207; 20 deaths); Firth penalized logistic regression				
Centenarian (≥100 years)	6.08 (2.28–16.05)	<0.001	4.87 (1.73–13.60)	0.003
ASA score, per 1-point increase	4.39 (2.10–9.94)	<0.001	4.03 (1.87–9.48)	<0.001
(B) 12-month mortality (*n* = 207; 78 deaths); logistic regression				
Centenarian (≥100 years)	9.67 (3.98–27.28)	<0.001	8.19 (3.16–24.22)	<0.001
Female sex	0.83 (0.46–1.49)	0.53	0.82 (0.43–1.60)	0.57
ASA score, per 1-point increase	2.53 (1.64–4.05)	<0.001	2.64 (1.54–4.73)	<0.001
CCI, per 1-point increase	1.12 (0.90–1.40)	0.32	0.88 (0.65–1.17)	0.38
Arthroplasty (vs. internal fixation)	1.63 (0.91–2.93)	0.10	1.12 (0.56–2.20)	0.75
Time from admission to surgery, per 24 h	1.20 (1.01–1.44)	0.044	1.23 (1.01–1.51)	0.039

Adjusted odds ratios (ORs) are mutually adjusted for all variables listed for each outcome. The 30-day model was fitted with Firth’s penalized likelihood, with profile penalized likelihood confidence intervals (CIs); unadjusted 30-day ORs were also estimated with Firth’s method. 12-month model: C-statistic 0.75; Hosmer–Lemeshow test *p* = 0.94 (no evidence of lack of fit). ASA, American Society of Anesthesiologists; CCI, Charlson Comorbidity Index (original version without age points). Sensitivity analyses are shown in Appendix A, Table A2.

## Data Availability

The data supporting the findings of this study are not publicly available due to privacy and ethical restrictions. The data may be made available from the corresponding author upon reasonable request and with permission from the relevant institutional authorities.

## Abbreviations

The following abbreviations are used in this manuscript:

ASA	American Society of Anesthesiologists
CCI	Charlson Comorbidity Index
ICU	Intensive care unit
SD	Standard deviation
IQR	Interquartile range
CI	Confidence interval
OR	Odds ratio

## Appendix A

**Table A1:** Thirty-day and 12-month mortality by age group.

Age Group (Years)	*n*	30-Day Mortality, *n* (%; 95% CI)	12-Month Mortality, *n* (%; 95% CI)
65–69	19	0 (0.0; 0.0–16.8)	5 (26.3; 11.8–48.8)
70–79	55	4 (7.3; 2.9–17.3)	13 (23.6; 14.4–36.3)
80–89	84	6 (7.1; 3.3–14.7)	30 (35.7; 26.3–46.4)
90–99	18	1 (5.6; 1.0–25.8)	5 (27.8; 12.5–50.9)
≥100	31	9 (29.0; 16.1–46.6)	25 (80.6; 63.7–90.8)
*p* for trend		<0.001	<0.001

95% confidence intervals (CIs) were calculated with the Wilson method; *p* for trend, Cochran-Armitage test. Estimates for age groups with few patients or events are imprecise and should be interpreted with caution.

**Table A2:** Sensitivity analyses.

Analysis and Variable	OR (95% CI)	*p*
(i) 30-day mortality, extended Firth model (*n* = 207; 20 deaths)		
Centenarian (≥100 years)	4.83 (1.56–15.40)	0.007
ASA score, per 1-point increase	3.48 (1.51–8.70)	0.003
CCI, per 1-point increase	1.12 (0.74–1.70)	0.58
Arthroplasty (vs. internal fixation)	1.20 (0.41–3.32)	0.73
Time from admission to surgery, per 24 h	1.01 (0.73–1.33)	0.93
(ii) 12-month mortality, Firth model (*n* = 207; 78 deaths)		
Centenarian (≥100 years)	7.34 (2.93–20.71)	<0.001
Female sex	0.82 (0.43–1.58)	0.56
ASA score, per 1-point increase	2.53 (1.49–4.46)	<0.001
CCI, per 1-point increase	0.89 (0.66–1.17)	0.40
Arthroplasty (vs. internal fixation)	1.12 (0.57–2.17)	0.74
Time from admission to surgery, per 24 h	1.22 (1.01–1.49)	0.040
(iii) Age as a continuous variable instead of centenarian status		
30-day (Firth): age, per 5 years	1.37 (1.10–1.72)	0.004
30-day (Firth): ASA score, per 1-point increase	4.24 (1.99–9.91)	<0.001
12-month: age, per 5 years ‡	1.48 (1.25–1.77)	<0.001
12-month: ASA score, per 1-point increase ‡	2.89 (1.65–5.25)	<0.001
12-month: time from admission to surgery, per 24 h ‡	1.23 (1.01–1.51)	0.043
(iv) Patients aged ≥90 years: centenarians (*n* = 31) vs. nonagenarians (*n* = 18)		
30-day mortality: 9/31 (29.0%) vs. 1/18 (5.6%), Fisher *p* = 0.070; centenarian, ASA-adjusted (Firth)	2.82 (0.49–29.32)	0.26
12-month mortality: 25/31 (80.6%) vs. 5/18 (27.8%), Fisher *p* < 0.001; centenarian, ASA-adjusted (Firth)	6.53 (1.70–27.84)	0.006
(v) 12-month mortality, primary model plus postoperative complications (*n* = 207; 78 deaths)		
Any postoperative complication	2.61 (1.28–5.40)	0.009
Centenarian (≥100 years)	9.09 (3.42–27.65)	<0.001
ASA score, per 1-point increase	2.57 (1.49–4.63)	0.001
Time from admission to surgery, per 24 h	1.28 (1.04–1.59)	0.021

OR, odds ratio; CI, confidence interval; ASA, American Society of Anesthesiologists; CCI, Charlson Comorbidity Index (original version without age points). ‡ Adjusted for sex, CCI and surgical procedure in addition to the variables shown. Firth models use profile penalized likelihood CIs. Complications are postoperative events; analysis (v) is presented as a secondary, descriptive association and not as a prognostic model.
